# Facilitated Chromium(VI) Transport across an Ionic Liquid Membrane Impregnated with Cyphos IL102

**DOI:** 10.3390/molecules24132437

**Published:** 2019-07-02

**Authors:** Francisco Jose Alguacil

**Affiliations:** Centro Nacional de Investigaciones Metalurgicas (CSIC), Avda. Gregorio del Amo 8, 28040 Madrid, Spain; fjalgua@cenim.csic.es

**Keywords:** chromium(VI), Cyphos IL102, membrane transport, recovery, ionic liquids, environment

## Abstract

Chromium(VI) is a well-known hazardous element, thus, its removal from aqueous sources is of a general concern. Among the technologies used for the removal of this type of toxic elements, liquid membranes are gaining in importance and the same has occurred with the use of ionic liquids, considered for many, due to their properties, as green solvents. Thus, the present work joined the three previous points, presenting an experimental study about the removal of chromium(VI) by the use of a liquid membrane operation which used the commercially available Cyphos IL102 ionic liquid as a carrier. The experimental variables included: the stirring speed applied to the feed and receiving solution (a key-parameter to gain maximum transport), acid, chromium(VI), sodium hydroxide and Cyphos IL102 concentrations in their various phases. Additionally, the performance of the present system was evaluated both against the presence of other metals in solution and other carriers. The experimental results confirmed that Cyphos IL102 is a good carrier for chromium(VI) transport and, thus, its removal from aqueous streams, and it also performed well in the presence of accompanying metals and against the performance of other commercially available carriers.

## 1. Introduction

Ionic liquids, or green solvents, are a group of salts with various anion-cation compositions, which are liquids at a temperature below 100 °C. Having some specific features, such as versatile solubility, non-volatility, physical and chemical stabilities, etc., make them a real alternative to hazardous volatile organic chemicals, especially solvents, though there are also serious concerns about the hazardousness of ionic liquids. Their applications do not end as substitutes of such solvents because, due to another important feature of these chemicals, i.e., the ability of their molecular structure to be tailored (task-specific ionic liquids) according to application requirements; they expand to catalysis, coordination chemistry, analytical chemistry, polymer materials, nanotechnologies, lithium-based batteries, etc., and also in separation processes of metals and other solutes [1,2,3,4,5,6,7,8,9,10,11,12,13,14,15,16].

In the case of the removal of metals present in aqueous solutions, chromium(VI) had found, and still has, a paramount interest due to its toxic and carcinogenic character [17]. Much had been written about the hazardousness of this element, upon ingesta, by humans, with a real episode of Cr(VI) contamination, during almost the 30 last year period of the 20th century, in a city located in the USA. As a result of this interest, researchers had developed a series of technologies in order to remove the element from the aqueous solutions containing in it. In many cases, ionic liquids have played a key-role in the success of the process, i.e., liquid–liquid extraction [18,19], ion exchange [20,21], adsorption [22,23], and liquid membranes [24,25], and very often the results obtained by the various authors are difficult to compare due to the different experimental conditions used, which, in the case of supported liquid membranes, included solid support, stirring speeds, carrier concentrations and type of organic diluent, etc.

Considering the case of liquid membranes technology, and particularly supported liquid membranes operation mode, such membranes emerged as a real alternative to the liquid–liquid extraction processing in the field of separation technologies. Metal transport through liquid membranes is governed by kinetics parameters instead of chemical equilibrium between the organic and aqueous phases, characteristic for liquid–liquid extraction operation; this feature is of great advantage for the concentration of metals, in the receiving or strip phase, as compared with liquid–liquid extraction. Other characteristic features of liquid membranes are: low energy input, the use of small amounts of organic phases in the operation, the possibility of the treatment of solutions with a low metal concentration, and that the extraction and stripping stages occurred simultaneously. The liquid membrane operation combines the kinetics and selectivity of liquid–liquid extraction and the simplicity of the membrane diffusion processes [26,27].

The present investigation joined the three focal points briefly mentioned above: (i) ionic liquids, (ii) chromium(VI) toxicity and the necessity of its removal from aqueous streams, and (iii) the usefulness of supported liquid membranes in this environmental field of interest. Cyphos IL 102 (trihexyl tetradecylphosphonium bromide) was used to remove different metals from aqueous solutions [28,29,30], and it is used, in the present work, as a carrier, since no data were apparent available about its use in the removal of Cr(VI)-bearing aqueous solution, broadening the knowledge about the performance of the Cyphos family of ionic liquids in this field, and also to continue the investigations that the present author is carrying out on the transport of Cr(VI) using different carriers. In the flat-sheet supported liquid membrane (FSSLM) technology used here, the carrier impregnates the membrane support, and different and important hydrodynamic and chemical operational variables affecting the transport of Cr(VI) across the membrane are investigated: the stirring speed applied to the feed and receiving phases, the composition of the feed, receiving and organic phases; also the selectivity of the system against the presence of other metals in the feed phase is evaluated. Further, the aim of this work, in terms of Cr(VI) transport, and better to compare the results obtained by different authors using different carriers (as it is said above, using different operational parameters), is to evaluate the performance of Cyphos IL102, against that of several industrial and potential Cr(VI) carriers, including a couple of ionic liquids, using the very same experimental conditions. In this investigation, these all important operational variables are deeply studied in order to optimize the transport operation of Cr(VI) from acidic liquid wastes, the investigation also aims to the evaluation of the mass transfer parameters.

## 2. Results and Discussion

Though there is not any specific information about the extraction of chromium(VI) by this ionic liquid, information about other systems [31,32], suggests that the transport of chromium(VI) through a supported liquid membrane impregnated with Cyphos IL102 can be attributed to the next reactions:

(1) $$R_{3}R\prime P^{+}Br_{m}^{-}+HCrO_{4_{f}}^{-}\to R_{3}R\prime P^{+}HCrO_{4_{m}}^{-}+Br_{f}^{-}$$

In this, and successive equations, f, m, and r refer to the feed, membrane, and receiving phases, respectively. Accordingly with the above reaction, the chromium(VI) species react with the ionic liquid in the feed-membrane interface, releasing bromide ions to the feed phase, then in the membrane-receiving solution interface, chromate ions are released to the receiving phase with formation of R_3_R′P^+^OH^−^ species in the membrane phase:

(2) $$R_{3}R\prime P^{+}HCrO_{4_{m}}^{-}+2OH_{r}^{-}\to R_{3}R\prime P^{+}OH_{m}^{-}+CrO_{4_{m}}^{2-}+H_{2}O$$

In the feed-membrane interface the next reaction occurs:

(3) $$R_{3}R\prime P^{+}OH_{m}^{-}+Br_{f}^{-}\to R_{3}R\prime P^{+}Br_{m}^{-}+OH_{f}^{-}$$

which regenerates the ionic liquid and Equation (1) is carried out again. When dichromate species are predominant in the feed phase (this occurs at chromium(VI) concentrations higher than 0.1 g/L), the next reactions also takes part (fully or partially if HCrO_4_^−^ and Cr_2_O_7_^2−^ co-exists in the feed phase) in the transport process:

(4) $$2R_{3}R\prime P^{+}Br_{m}^{-}+Cr_{2}O_{7_{f}}^{2-}\to {\left(R_{3}R\prime P^{+}\right)}_{2}Cr_{2}O_{7_{m}}^{2-}+2Br_{f}^{-}$$

(5) $${\left(R_{3}R\prime P^{+}\right)}_{2}Cr_{2}O_{7_{m}}^{2-}+4OH_{r}^{-}\to 2R_{3}R\prime P^{+}OH_{m}^{-}+2CrO_{4_{m}}^{2-}+H_{2}O$$

Equations (3) and (1) follow again. There is the possibility that the transport process occurs via the transport of ion-paired species, thus, in the case of HCrO_4_^−^ and in the feed-membrane interface:

(6) $$R_{3}R\prime P^{+}Br_{m}^{-}+H_{f}^{+}+HCrO_{4_{f}}^{-}\to \left(R_{3}R\prime P^{+}Br^{-}\right){\left(H_{2}CrO_{4}\right)}_{m}$$

whereas in the membrane-receiving solution interface:

(7) $$\left(R_{3}R\prime P^{+}Br^{-}\right){\left(H_{2}CrO_{4}\right)}_{m}+2OH_{r}^{-}\to R_{3}R\prime P^{+}Br_{m}^{-}+CrO_{4_{r}}^{2-}+2H_{2}O$$

which regenerates the ionic liquid and Equation (1) occurs again. Thus, the transport of chromium(VI) by this ionic liquid can be attributed to an anion-exchange process, Equations (1) and (4), being the corresponding stoichiometry dependent of the chromium(VI) concentration in the feed phase, and also/or by the transport of ion-paired species, Equation (6), and similarly in the case of the presence of Cr_2_O_7_^2−^ species in the feed solution.

### 2.1. Influence of the Stirring Speed of the Feed Solution on Cr(VI) Transport

In this batch liquid membrane operation, the stirring speed of the feed solution may have a key-role in the transport process, thus, this experimental variable was first investigated by the use of feed solutions containing 0.01 g/L Cr(VI) and 0.01 M HCl and water as the receiving phase, whereas the membrane phase was of 0.12 M Cyphos IL102 in Solvesso 100. The results from these series of experiments were summarized in Table 1, and the overall mass transfer coefficient K was calculated according to the next relationship (details about the equation are given in Section 3):

(8) $$\ln\frac{{\left[Cr\right]}_{f,t}}{{\left[Cr\right]}_{f,0}}=-\frac{A}{V}Kt$$

From the results shown in Table 1, it can be seen that the value of K increased with the increase of the stirring speed, applied to the feed phase, up to 1000 min^−1^ and then remained constant; thus, from 1000 min^−1^ the aqueous feed layer reached a minimum with maximized the value of the overall mass transfer coefficient, minimized the resistance to mass transfer, and apparently, the transport process was controlled by diffusion in the stagnant film of the feed solution. Thus, at 1000 min^−1^, a limiting value of K is reached and:

(9) $$K_{\lim}=\frac{D_{Cr,f}}{d_{f}}$$

D_Cr,f_ being the feed diffusion coefficient of the chromium-containing species (averaging 6 × 10^−4^ cm^2^/min) and d_f_ the minimum thickness of the feed layer under the present experimental conditions. From this system, d_f_ was estimated as: 2.1 × 10^−3^ cm. Table 1 showed that at a stirring speed of 1200 min^−1^ the value of K decreased, this is attributable to a displacement of carrier phase from the membrane pore by high turbulence caused by vigorous stirring.

Since:

(10) $$J_{\lim}=K_{\lim}{\left[Cr(VI)\right]}_{f,0}$$

A limiting flux value was estimated as 5.4 × 10^−8^ mol/cm^2^ min. Under this limiting condition:

(11) $$J_{\lim}=\frac{D_{m}\left[CyphosIL102\right]}{nd_{m}}$$

and the value of the membrane diffusion coefficient, D_m_, was estimated as 5.6 × 10^−6^ cm^2^/min, considering *n* = 1 (Cyphos IL 102 stoichiometric factor in Equation (1), and d_m_ of 12.5 × 10^−3^ cm (see Section 3). Furthermore, the diffusion coefficient of the chromium(VI) species in the bulk membrane phase, D_m,b_, can be also estimated as:

(12) $$D_{m,b}=\frac{D_{m}\tau^{2}}{\varepsilon }$$

τ and ε being the membrane tortuosity and porosity, respectively (see Section 3). Thus, D_m,b_ was 2.1 × 10^−5^ cm^2^/min. The comparison of D_m_ and D_m,b_ values for the present system, showed that D_m_ value is lower than that of D_m,b_, this being attributed to the diffusional resistance caused by the membrane.

The stirring speed of 1000 min^−1^ in the feed phase was used in all the subsequent experiments.

### 2.2. Influence of the Receiving Phase Composition and Stirring Speed on Chromium (VI) Transport

Besides water, sodium hydroxide solutions were used to investigate their influence on chromium(VI) transport, and, more importantly, the recovery of the element in the receiving phase. In this series of experiments the same experimental conditions as above were maintained constant, whereas 0.1, 0.5 or 1 M NaOH solutions were used as receiving phases. The results from these experiments are shown in Table 2.

The composition of the receiving phase had no effect on the K value, but it had a remarkable influence in the case of the percentage of chromium(VI) recovered in the receiving phase, especially when sodium hydroxide solutions were used instead of water. From this Table it can be also concluded that the increase of the OH^−^ concentration in the receiving solution had a negligible influence on K value. The independence of the metal transport on the OH^-^ concentration might be an indication that in the present system the R_3_R´P^+^OH^−^ species, Equations (2) and (5), did not contribute apparently to the transport process. Further transport experiments were conducted using a 0.5 M NaOH solution as receiving phase.

In the case of the investigation of the influence of the stirring speed applied to the receiving phase on K value, all the experimental conditions used above were fixed except the stirring speed of the receiving phase which was varied between 400 and 800 min^−1^. This experiments showed that the variation of the stirring speed in the above range, had a negligible effect both on the K value (0.28 cm/min) and on the percentage of chromium recovered in the receiving phase (58.3% and 58.6% for the stirring speeds of 400 and 800 min^−1^, respectively). Thus, 400 min^−1^ was selected throughout all further experiments conducted in this investigation.

### 2.3. Influence of HCl Concentration in the Feed Solution on Cr(VI) Transport

The effect of the acid concentration on chromium(VI) transport was evaluated at different HCl concentration ranging from 0.01 to 10 M. Table 3 summarized the results from these experiments.

The results presented in Table 3 evidence that as the acidity of the aqueous feed phase increased, there is a marked decrease in chromium(VI) transport. Thus, from HCl concentrations above 2.5 M Equation (1) has a negligible contribution to the metal transport; also, the increase of the HCl concentration is accompanied by an increase of I (ionic strength), and this increase has a negative influence on the transport of chromium(VI) by Cyphos IL102. Additionally, an increase in the ion concentrations, due to the increase of I, in the solution (crowding effect) may have an influence on a given system.

### 2.4. Influence of the Initial Chromium(VI) Concentration in the Feed Phase on the Overall Mass Transfer Coefficient Value

**Figure 1:**
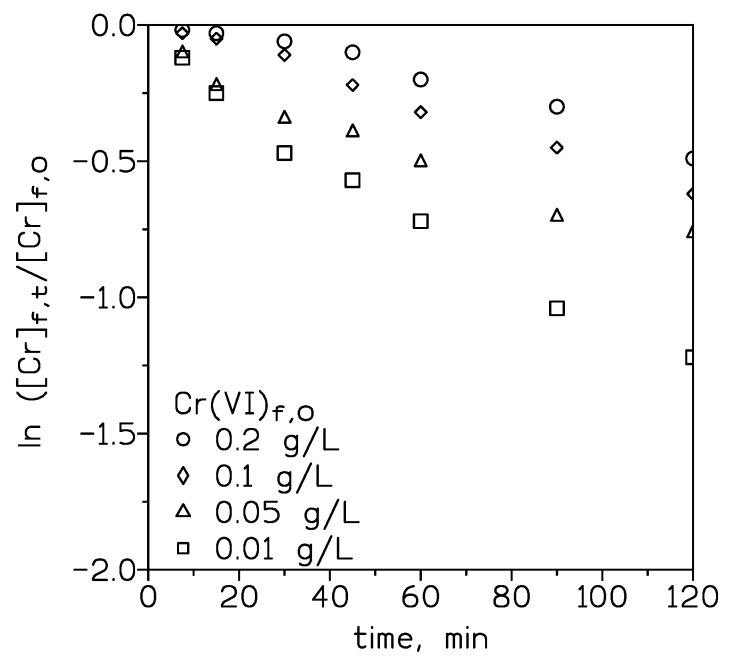
Influence of the initial chromium(VI) concentration in the feed solution on metal transport. Feed phase: 0.01 g/L Cr(VI) and HCl. Stirring speed: 1000 min^−1^. Membrane phase: 0.12 M Cyphos IL102 in Solvesso 100. Membrane support: Durapore HVHP. Receiving phase: 0.5 M NaOH. Stirring speed: 400 min^−1^.

A range of experiments were conducted to investigate the effect that the initial chromium(VI) concentration in the feed solution had on the K value. The results from these experiments were shown in Figure 1, plotting ln([Cr]_f,t_/[Cr]_f,0_) versus time. In this Figure two zones can be defined. At longer period times, and from the beginning of the experiment in the case of the chromium concentrations of 0.01 and 0.05 g/L, a straight line was defined and the system responded well to Equation (8), with corresponding overall mass transfer coefficients values showed in Table 4. It is shown that these values decreased as the initial chromium concentration increased in the feed solution. For each, chromium(VI) concentration the metal flux can be estimated as:

(13) $$J=K{\left[Cr(VI)\right]}_{f,0}$$

and the values were shown in Table 4. It was seen that the flux value increased sharply with the increase of the initial metal concentration of the feed solution, and then seemed to became independent of the initial chromium(VI) concentration. The initial increase of the flux with the metal concentration was in accordance with that was expressed in Equation (13), whereas the trend to a constant flux value was attributable to a saturation of the membrane pores, resulting in a lower effective membrane area towards the metal transport.

At short elapsed times and for chromium concentrations above 0.1 g/L, and from data presented in Figure 1, an induction period was observed. In these conditions, the metal transport is controlled by diffusion and Equation (8) is not valid, the next equation then must be considered:

(14) $${\left[Cr\right]}_{f,t}={\left[Cr\right]}_{f,0}-\frac{{\left[CyphosIL102\right]}_{m}D_{org}A}{nd_{m}V}$$

where D_org_ is the membrane diffusion coefficient, and n is the number of carrier molecules involved in chromium transport. [Cr]_f,t_ and [Cr]_f,0_ had the significance showed in Section 3.

Since from 0.1 g/L Cr(VI) in the aqueous solution, Cr_2_O_7_^2−^ becomes predominant in the feed phase, Equation (4) was only considered in chromium transport, and thus, *n* = 2 in Equation (14). A plot of [Cr]_f,t_ versus t allowed D_org,_ to be estimated, which in the present case was 2.5 × 10^−5^ cm^2^/min, considering a carrier concentration of 0.12 M. It should be noted here, that D_org_ value is independent of the initial metal concentration in the feed phase, since this concentration term does not appeared in the extreme right term of Equation (14). Furthermore, the initial overall mass transfer coefficient, K_0_, can be estimated from the next relationship:

(15) $$K_{0}=\frac{{\left[CyphosIL102\right]}_{m}D_{org}}{{\left[Cr\right]}_{f,0}nd_{m}}$$

and K_0_ values were estimated as 6.3 × 10^−2^ and 3.1 × 10^−2^ cm/min for the chromium concentrations of 0.1 and 0.2 g/L, respectively, and considering again a 0.12 M Cyphos IL 102 concentration and *n* = 2.

Results showed in Table 4 also indicated that under the present experimental conditions, the percentage of chromium(VI) recovered in the receiving solution was dependent on the initial metal concentration in the feed phase.

### 2.5. Influence of Carrier Concentration on Chromium(VI) Transport

In this set of experiments the influence of the Cyphos IL102 concentration on metal transport was evaluated using carrier concentrations ranging from 0.003 to 0.12 M; as usual, the carrier was dissolved in Solvesso 100. Table 5 presents the data derived from the experiments.

From these result it was shown that the increase of the carrier concentration solution impregnating the membrane pores had a positive effect on metal transport, increasing the value of the overall mass transfer coefficient (and the percentage of metal transport) from 0.21 (56.4%) to 0.28 (70.5%) cm/min for the range of carrier concentration investigated. It is also shown in the table that the variation of these carrier concentrations had a negligible effect on the percentage of chromium recovered in the receiving solution.

### 2.6. Contribution of Mass Transfer Resistances to the Overall Chromium(VI) Transport Process

It is described in the literature [33], that the equilibrium and diffusional parameters involved in the transport process, can be combined in an equation of the form:

(16) $$\frac{1}{K}=\Delta_{f}+\frac{\Delta_{m}}{C}$$

where C is a parameter, involved in the extraction or transport process, which considers the extraction equilibrium constant or constants and the concentration of the species which participated within the process. Δ_f_ and Δ_m_ were the transport resistances in relation to diffusion by the feed boundary layer and the membrane, respectively.

In a transport process, the overall mass transfer resistance was the sum of the different resistances participating in the process and, thus, Equation (16) was rewritten as:

(17) $$R=R_{f}+R_{m}$$

Table 6 shows the contribution of these various resistances to the overall resistance.

### 2.7. Transport of Cr(VI) from Cr(VI)-Metal Binary Solutions

It seemed of interest to compare the performance of the present Cr(VI)-Cyphos IL102 system when other metals were present in the feed solution. Thus, a series of tests was conducted using binary mixtures of Cr(VI) in the presence of Cu(II), Fe(III), Co(II), Cr(III), Mn(VII) (in the form of MnO_4_^-^) and Au(III) (in the form of AuCl_4_^−^). Then, the feed solution contained mixtures of the elements in an equimolar 1.9 × 10^−4^ M concentration in 0.01 M HCl. The results from these series of experiments are shown in Table 7.

These results showed that chromium(VI) was transported quantitatively from other metals presents in the feed solution except from Mn(VII) and Au(III). Furthermore, Mn(VII) was transported preferably to Cr(VI) since β_Cr(VI)/Mn(VII)_ was estimated as 0.23. β, or the separation factor, defines if two elements can be separated by means of a separation technology (liquid–liquid extraction, ion exchange, adsorption, liquid membranes). In the case of liquid membranes, the separation factor is defined as:

(18) $$\beta_{M1/M2}=\frac{K_{1}}{K_{2}}$$

where K are the corresponding overall mass transfer coefficient values. Additionally, this relationship can be expressed in terms of metal permeability or P_M_. Thus, if β > 1 the metals can be separated, though this value gives no indication about the easiness of the separation.

The comparison of the K values presented in Table 7 with respect to the K value for the single transport of Cr(VI), indicated that this element was transported less favourably (lower K value) in the presence of the accompanying metal in the feed solution. This was a logical issue in the case of chromium(VI) transport in the presence of manganese(VII) and gold(III), since both elements were transported across the membrane containing Cyphos IL102, and there was a competition to bond with the ionic liquid, but it seemed less logical when other non-transportable elements are present in the feed phase. The explanation to this issue is the crowding or population effect that makes that the value of the overall mass transfer coefficient (or permeability coefficient) of a given metal decreases in the presence of other elements in the aqueous solution. This is not a generalized issue and its effect must be experimentally determined for each binary, ternary, etc., system.

Regarding the recovery of chromium in the receiving phase, experimental data indicated that in all the systems showed in Table 7, the percentage of Cr(VI) recovered was around 58% after 2 h running time, a value which compared extremely well with the yield (58.3%) when single Cr(VI) was transported across the membrane impregnated with Cyphos IL102.

### 2.8. Transport of Cr(VI) using Different Carriers

Following the investigations on the system Cr(VI)-Cyphos IL102 and its performance on chromium(VI) transport, this section presents experimental data about the transport of the metal using the same membrane HVHP support impregnated with different carriers dissolved in Solvesso 100. It should be noted here, that all the carriers used in this experimentation are classified as industrial, that is, they are not mere laboratory-developed compounds. The best transport results were obtained when solutions of Amberlite LA2 and Aliquat 366 in Solvesso are used to impregnate the support, whereas the worst K values resulted from the use of Cyanex 471× and TBP as carriers for Cr(VI) transport. In the case of ionic liquid compounds, the series followed the order Aliquat 336 > Cyphos IL101 > Cyphos IL102, whereas in the case of amines the order found was: secondary (Amberlite LA2) > tertiary (Hostarex A324) > primary (Primene JMT). A point to consider here is that amines are potential precursors of ionic liquids when contacted with the suitable concentration of a mineral acid in an aqueous solution, these reactions resulting in compounds with RNH_3_^+^X^−^, R_2_NH_2_^+^X^−,^ and R_3_NH^+^ stoichiometries for primary, secondary, and tertiary amines, respectively, whereas X denotes the counter-anion of the mineral acid. In the case of neutral organic derivatives of the phosphoric acid, the results of Table 8 indicate that the apparent Cr(VI) transport order is: phosphine oxide>phosphonic ester > phosphoric ester.

Results from Table 8 also showed that the recovery of Cr(VI) in the receiving solution was very dependent of the carrier used to impregnate the membrane support, but in all the cases the recovery found was near or above 50%, except in the case of DBBP, which was around 40%.

### 2.9. Transport of Cr(VI) under two Types of Liquid Membrane Operations

The performance of the flat-sheet supported liquid membrane (FSSLM) was compared with other membrane technology named pseudo-emulsion membrane with strip dispersion (PEMSD), see Section 3. The experiments with the PEMSD technology were conducted using aqueous solutions containing 0.01 g/L or 0.05 g/L Cr(VI) in 0.01 M HCl medium, and strip phases of 0.5 M NaOH. The membrane support was of a HVHP support impregnated with a 0.007 M Cyphos IL102 in Solvesso 100 solution. The results derived from these experiments are summarized in Table 10.

These results show that the change in the membrane technology did not altered the transport of Cr(VI) from the feed solution to the membrane phase. The most important feature of this PEMSD smart technology was that the percentage of Cr(VI) recovered in the receiving solution improved quite substantially (95.9% against 58.8% for FSSLM). This undoubtedly was the result of the formation of the pseudo-emulsion phase between the organic and receiving phases, and the intimate contact between droplets of these two phases in the cell chamber, which favored the metal transfer from the Cr(VI)-loaded organic solution to the receiving solution. Additionally, the concentration of the metal in the receiving solution increased with respect to that of the initial feed solution (0.013 g/L against 0.01 g/L, respectively) due to the variation of volumes used in this type the experimentation: V_feed_ = 200 mL versus V_receiving_ = 100 mL. As it was expected, the K value of the more concentrated Cr(VI) solution (0.05 g/L) decreased with respect to the value yielded with the 0.01 g/L feed solution.

### 2.10. Post-Treatment of the Cr(VI)-Bearing Receiving Solutions

A post-treatment of the receiving solution containing chromium(VI) was derived from previous results in the literature [34]. Thus, a receiving solution containing circa 0.02 g/L Cr(VI) in 0.5 M NaOH, was treated drop-wise with a 5 g/L hydrazine sulphate solution; almost near the addition of the first drops, chromium(VI) reduced to the (III) oxidation state, this reduction was completed after the addition of 1 mL of the hydrazine solution to 100 mL of the alkaline Cr(VI) solution:

(19) $$4CrO_{4}^{2-}+7H_{2}O+\frac{3}{2}N_{2}H_{6}^{2-}\to 4Cr{\left(OH\right)}_{4}^{-}+7OH^{-}+\frac{3}{2}N_{2}$$

Following this post-treatment, the still hazardous Cr(VI) receiving solution were transformed to the less toxic Cr(III) solution, facilitating its handling and possible storage; even, the yielding of a Cr(III)-bearing pigment can be achieved by a pH-controlled precipitation process.

## 3. Materials and Methods

Cyphos IL 102 (trihexyl tetradecylphosphonium bromide) was supplied from Cytec Ind. (Woodland Park, NJ, USA), whereas other extractants or carriers used in this work were obtained from different sources, these and their chemical compositions are shown in Table 9. Cyphos IL 102 and all those carriers were used as supplied by the manufacturer. Solvesso 100 (Iberia Chem., Madrid, Madrid Community, Spain) is an aromatic (99%) organic diluent and also used as such. All other chemicals used were of G.R grade. The support used in this investigation was Durapore HVHP1400 with 75% nominal porosity (ε), 12.5 × 10^−3^ thickness (d_m_), 0.45 μm pore size and 1.67 tortuosity (τ).

The use of an organic diluent as Solvesso 100 was not a drawback for the present investigation because Solvesso 100 is an industrially available diluent which comply all the security norms. The use of the diluent was also useful because: (i) it serves to accommodate the range of the carrier concentrations to each particular use; in this type of separation process the cost of the carrier is the most important budget item, and it made little sense to use an excess of carrier concentration with no use; (ii) it allowed a decrease of the viscosity of the organic phase, as ionic liquids are particularly viscous and this property is a real drawback for any supported liquid membrane operation (like the use here or with hollow fiber modules), since it was a general and experimental finding norm, that an increase in the organic solution viscosity produced an immediate decrease in the transport of a given metal (as an exception to any rule, this result was no found in the present work due to the low range of Cyphos IL102 concentrations used in the experimentation).

Flat-sheet supported liquid membrane experiments were carried out in a two-compartment cell described elsewhere, as is the experimental procedure [35], whereas pseudo-emulsion membrane strip dispersion tests were conducted in the same cell, but in this case, in the receiving compartment, the receiving phase was substituted by a given volume of an organic phase similar to the one which impregnated the solid support and a given volume of the receiving phase. In the present investigation, the volumes of both the organic and the receiving solutions were of 100 mL each. The mixture of the two phases was contacted by agitation of the phases, the receiving phase being dispersed into the organic solution [36]. After stopping the agitation, the phases were separated by gravity, the time required for this separation being no more than 3 min. This previous procedure was similar to that followed in hollow fiber modules using this strip phase dispersion operation [37,38].

All the above experiments were conducted at 20 °C.

Chromium(VI) and other metals in the aqueous solutions were analyzed by atomic absorption spectrometry, and the transport of the metals was computed using the expression showed in Equation (8) (see Section 2), for the case of Cr(VI). In this Equation (8), A is the area of the support used in this work (11.3 cm^−2^), V is the volume of the feed phase (200 mL), t is the elapsed time, and [Cr(VI)]_f,t_ and [Cr(VI)]_f,0_ are the chromium concentrations in the feed at time t and time 0, respectively.

The percentage of chromium(VI) recovery in the receiving solution was calculated as:

(20) $$\%=\frac{{\left[Cr\left((VI\right)\right]}_{r,t}x100}{{\left[Cr(VI)\right]}_{f,0}-{\left[Cr\left(VI\right)\right]}_{f,t}}$$

where [Cr(VI)]_r,t_ was the chromium concentration in the receiving solution at time t.

## 4. Conclusions

Durapore HVHP1400 support impregnated with Cyphos IL102 ionic liquid is very efficient for the transport of Cr(VI) from HCl solutions. This effectiveness is of the same order than that was obtained with the similar Cyphos IL101 ionic liquid, as the values of the overall mass transfer coefficients resulted within the present investigation shown: 0.28 against 0.30 cm/min for IL102 and IL101, respectively, whereas the recovery of Cr(VI) in the receiving solution is even higher (58.3% for IL102, and 50.1% for IL101). At a stirring speed in the feed phase of 1000 min^−1^, a limiting value of 0.28 cm/min for the overall mass transfer coefficient is obtained and the transport process is controlled by diffusion in the feed phase stagnant film. Cr(VI) is selectively transported from HCl solutions using a supported liquid membrane impregnated with Cyphos IL102 over Cu(II), Co(II), Fe(III) and Cr(III), allowing to the selective speciation of Cr(VI)-Cr(III) from aqueous solutions. Against many transport systems, time dependent overall mass transfer coefficient is obtained at higher Cr(VI) concentrations (0.1 and 0.2 g/L) in the feed solutions and short running times. In these cases, initial values of the overall mass transfer coefficient are found to be 6.3 × 10^−2^ and 3.1 × 10^−2^ cm/min for the above metal concentrations, respectively. Under various experimental conditions, stirring speed of the feed phase, HCl and Cr(VI) concentrations in the same phase and carrier concentration in the membrane phase, it is concluded that the diffusion by the aqueous feed boundary layer contributed in a major extend to the overall transport process. An advanced membrane technology such as PEMSD (pseudo-emulsion membrane strip dispersion), though does not improve the value of the overall mass transfer coefficient greatly increases the percentage of Cr(VI) recovered in the receiving solution. Further transport improvement of the overall process, by the use of hollow fiber modules, is expected.

## Figures and Tables

**Table 1:** Values of K at various stirring speeds of the feed phase.

Stirring Speed, min^−1^	K, cm/min
400	0.12
800	0.25
1000	0.28
1200	0.18

Membrane support. Durapore HVHP. Stirring speed of the receiving phase: 400 min^−1^.

**Table 2:** Values of K using different receiving phases.

Receiving Phase	K, cm/min	^a^ % Cr(VI) Recovered in the Receiving Phase
Water	0.28	13.4
0.1 M NaOH	0.28	56.1
0.5 M NaOH	0.28	58.3
1 M NaOH	0.28	60.6

Membrane support: Durapore HVHP. Stirring speed of the receiving phase: 400 min^−1^. ^a^ After 2 h.

**Table 3:** Influence of the HCl concentration in the feed phase on the K value.

HCl, M	K, cm/min
0.01	0.28
0.1	0.28
0.5	0.27
1	0.27
2.5	0.25
5	0.13
10	0.04

Feed phase: 0.01 g/L Cr(VI) and HCl. Stirring speed: 1000 min^−1^. Membrane phase: 0.12 M Cyphos IL102 in Solvesso 100. Membrane support: Durapore HVHP. Receiving phase: 0.5 M NaOH. Stirring speed: 400 min^−1^.

**Table 4:** Overall mass transfer values at various initial Cr(VI) concentrations.

(Cr(VI))_f,0_, g/L	K, cm/min	J × 10^7^, mol/cm^2^ min	^a^ % Cr(VI) Recovered in the Receiving Phase
0.01	0.28	0.53	58.3
0.05	0.21	2	57.0
0.1	0.12	2.3	43.5
0.2	0.07	2.7	21.1

Experimental conditions as in Figure 1. ^a^ After 2 h.

**Table 5:** K values at various carrier concentrations.

(Cyphos IL102), M	K, cm/min	^a^ % Cr(VI) Recovered in the Receiving Phase
0.003	0.21	57.1
0.007	0.23	58.8
0.015	0.23	61.3
0.03	0.24	59.2
0.06	0.25	56.8
0.12	0.28	58.3

Feed phase: 0.01 g/L Cr(VI) and HCl. Stirring speed: 1000 min^−1^. Membrane phase: 0.12 M Cyphos IL102 in Solvesso 100. Membrane support: Durapore HVHP. Receiving phase: 0.5 M NaOH. Stirring speed: 400 min^−1^. ^a^ After 2 h.

**Table 6:** Contribution of R_f_ and R_m_ to the overall chromium(VI) transport process.

Experimental Condition	R, min/cm	R_f_, min/cm	%R_f_^0^	%R_m_^0^
Stirring speed	8.33–3.57	3.57	43–100	57–0
HCl concentration	3.57–25	3.57	100–14	0–86
Cr(VI) concentration	3.57–12.5	3.57	100–29	0–71
Carrier concentration	4.76–3.57	3.57	75–100	25–0

In this Table, the percentage of the contribution of the fractional resistance to the overall process, %R_f_^0^ and %R_m_^0^, was estimated for each experimental condition, it was concluded that the diffusion by the aqueous feed boundary layer contributed in a major extend to the overall transport process.

**Table 7:** K values in the transport of Cr(VI) from binary mixtures.

System	K_Cr_, cm/min	K_M_, cm/min
Cr(VI)	0.28	-
Cr(VI)-Cu(II)	0.24	Co(II) not transported
Cr(VI)-Fe(III)	0.25	Fe(III) not transported
Cr(VI)-Co(II)	0.22	Co(II) not transported
Cr(VI)-Cr(III)	0.18	^a^ Cr(III) not transported
Cr(VI)-Mn(VII)	0.15	0.65
Cr(VI)-Au(III)	0.18	0.15

Feed phase stirring speed: 1000 min^−1^. Membrane phase: 0.12 M Cyphos IL102 in Solvesso 100. Membrane support: Durapore HVHP. Receiving phase: 0.5 M NaOH. Stirring speed: 400 min^−1^. ^a^ Result derived from a previous experiment using a single Cr(III) feed solution.

**Table 8:** Transport of Cr(VI) using different carriers.

Carrier	Active Group	K, cm/min	^a^ % Cr(VI) Recovered in the Receiving Phase
Cyphos IL102	R_3_RP^+^Br^-^	0.28	58.3
DBBP	(RO)_2_RPO	0.08	39.4
Cyanex 471×	R_3_PS	0.05	52.4
Cyanex 921	R_3_PO	0.09	50.6
Cyphos IL101	R_3_R´P^+^Cl^−^	0.30	50.1
TBP	(RO)_3_PO	0.04	51.8
Primene JMT	RNH_2_	0.18	61.4
Cyanex 923	R_3_PO	0.16	70.0
Hostarex A324	R_3_N	0.27	54.4
Aliquiat 336	R_3_R´N^+^Cl^-^	0.35	48.4
Amberlite LA2	R_2_NH	0.37	51.3

Feed phase: 0.01 g/L Cr(VI) in 0.01 M HCl. Stirring speed: 1000 min^−1^. Membrane phase: HVHP support impregnated with 0.12 M carrier in Solvesso 100. Receiving phase: 0.5 M NaOH. Stirring speed: 400 min^−1^. ^a^ After 2 h. R and R′ different alkyl chains (see Table 9).

**Table 9:** Chemical composition and source for the carriers.

Carrier	Chemical Composition	Source
Cyphos IL102	Trihexyl tetradecylphosphonium bromide	Cytec Ind.
DBBP	Dibutyl butylphosphonate	Albright and Wilson
Cyanex 471X	Tri-isobutyl phosphine sulphide	Cytec Ind.
Cyanex 921	Tri-n-octyl phosphine oxide	Cytec Ind.
Cyphos IL101	Trihexyl tetradecylphosphonium chloride	Cytec Ind.
TBP	Tri-n-butyl phosphate	Fluka
Primene JMT	mixture of t-alkylprimary amines	Rohm and Haas
Cyanex 923	mixture of tri-n-alkyl phosphine oxides	Cytec Ind.
Hostarex A 324	Tri-iso-octylamine	Hoechst
Aliquiat 336	Tri-octyl methylammonium chloride	Fluka
Amberlite LA2	Secondary alkylamine	Fluka

**Table 10:** Transport of Cr(VI) using FSSLM and PEMSD technologies.

Membrane Technology	K, cm/min	^a^ % Cr(VI) Recovered in the Receiving Phase
^b^ FSSLM	0.23	58.8
^b^ PEMSD	0.22	95.9
^c^ PEMSD	0.18	80.0

^a^ After 2 h. ^b^ Feed phase: 0.01 g/L Cr(VI) in 0.01 M HCl. ^c^ Feed phase: 0.05 g/L Cr(VI) in 0.01 M HCl.
